# A mathematical theory of the transcription repression (TR) therapy of cancer – whether and how it may work

**DOI:** 10.18632/oncotarget.16957

**Published:** 2017-04-08

**Authors:** Yuxin Chen, Haijun Wen, Chung-I Wu

**Affiliations:** ^1^ State Key Laboratory of Bio-control, School of Life Science, Sun Yat-Sen University, Guangzhou, China; ^2^ Beijing Institute of Genomics, Chinese Academy of Sciences, Beijing, China; ^3^ Department of Ecology and Evolution, University of Chicago, Chicago, Illinois, USA

**Keywords:** cancer therapy theory, transcription repression, transcription addiction, actinomycin D, evolution of cancer

## Abstract

Transcription repression (TR) therapy of cancer has been widely discussed. Here, TR refers to global repression of transcription rather than specific targeting of cancer-causing genes such as MYC. TR drugs inhibit transcription by binding to the transcribed DNA or to RNA polymerase; for example, actinomycin D has been extensively used in research and therapy to shut down transcription globally [1–7]. As proliferating cells demand a high rate of transcription, restricting transcript production could be effective in slowing down cell proliferation. However, TR also deprives other less proliferative cells of new transcripts, thus leading to substantial toxicity [1, 8, 9]. We now develop a mathematical theory to exploit the greater demand for transcription in highly proliferating cells. A new strategy, referred to as the TRR (transcript repression-recovery) model, would insert a recovery phase to allow the more slowly proliferating cells to recover. It is most effective to have strong blocking for a short period (a few hours) followed by a longer recovery phase in each cell cycle. Hence, TRR can potentially achieve selective killing of cells based on their global transcription needs but precise fine-tuning is necessary.

## INTRODUCTION

There have been many proposals for using transcription repression (TR) therapy on cancers. While TR therapies often broadly include the repression of specific cancer driver genes, such as MYC, the underlying molecular bases for any cancer type are fairly heterogeneous as revealed by the cancer genomic and transcriptomic data [10–12]. Here, a more general mechanism of global transcription repression will be considered. In this mechanism, TR may target DNA, RNA, RNA polymerase I or II or various transcription complexes [13–21].

The efficacy of the TR treatment depends on how it affects transcript abundance across all genes. Growing cell mass needs more transcripts than non-dividing or slowly dividing cells. We ask how TR therapies can work against fast-dividing cells by either causing cell death or pushing cells out of active division without damaging other cells. TR falls into two major categories depending on whether the rate of transcription and transcript degradation are cell cycle-dependent. For most mRNAs, degradation and Pol II-directed transcription are not strongly influenced by cell cycles. In contrast, rRNA synthesis directed by Pol I increases greatly when cells enter the cell-division cycles [14, 22–25]. Treatment strategies should be very different for these two mechanisms. This analysis will focus on the simpler mRNA synthesis that is not affected by cell cycles. The more complex cell cycle-dependence of rRNA production will be addressed in a subsequent study.

While TR could be applied to inhibit cell proliferation, it can be toxic to normal and less-rapidly dividing cells as well. Actinomycin D (or dactinomycin), which has been widely used in molecular biology laboratories to repress transcription, is one such example [1–9]. The mathematical theory developed below aims at removing the toxicity to slower-dividing cells while preserving the potency of TR drugs against the more rapidly-dividing ones.

### The TRR (transcription repression-recovery) model and analysis

#### Overview

The model of TR therapy analyzed here is an attempt at disrupting faster dividing cells while sparing slowly dividing or resting ones. It has two main features. First, the proliferation rate of mammalian cells is assumed to be limited by their transcription capacity. Second, transcription repression is followed by a short period of recovery within each cell cycle. The model is referred to as the TRR (transcription repression-recovery) model.

We now elaborate on the first feature. When cells divide every day for 10 days and increase in number by 1000 fold (~2^10^), the demand for transcripts should be 1000 fold as well. This demand might be a limiting factor of mammalian cell proliferation. For example, the doubling-time in long term cell cultures often reaches a limit of ~ 20 hours, despite the advantage of faster divisions. This limit is not set by DNA replication since cells that divide without much transcription (as in early embryos) can proceed faster. Furthermore, the S phase in cell cycles generally accounts for less than 1/3 of the entire process, suggesting that transcription in the longer G1 + G2 phases are more likely to be the limiting factor. Given the average half-life of mammalian mRNAs (6–8 hours) [26, 27], it would take >16 hours to synthesize the needed new transcripts. Hence, a cell cycle of 20 hours is probably close to the limit if we include the time needed for the S and M phases. If transcription of mRNAs is the rate-limiting step of cell division, TR may handicap rapidly proliferating cells more severely.

The second feature of the TRR model is the inclusion of a recovery phase that may reduce, or even eliminate, the toxicity to slowly proliferating cells. The relative length of the repression vs. recovery phase will be a key parameter of the strategy.

### Dynamics of mRNA changes in resting vs. dividing cells

Let *X(t)* be the number of transcripts of an average gene at time *t* within a single cell. The change in a time unit of *X(t)* is given by

$$\frac{\partial X}{\partial t}=b-\text{dX}$$ Eq. (1)

where *b* is the transcription rate and *d* is the decay rate. Solving Eq. (1), we obtain

$$X_{t}=\frac{b}{d}-\left(\frac{b}{d}-X_{0}\right)e^{-\text{dt}}$$ Eq. (2)

When *t* approaches infinity, *X(t)* reaches the equilibrium, *X*_eq_, when $\frac{\partial X}{\partial t}=0$. Thus,

X_eq_ = b/d

*X_eq_* can be measured by RNAseq and d is obtained by the time-course measurements when all transcription is repressed, i.e., *b* = 0. With *b* = 0, Eq. (2) is reduced to

$$X_{t}=X_{0}e^{-\text{dt}}$$ Eq. (2.1)

where *X(0)* is usually equal to *X_eq_* in the beginning of the measurement.

In resting cells, *X(t)* approaches *X_eq_* and remains unchanged unless perturbed. For dividing cells, *X(t)* repeatedly decreases as a result of cell division. If most of the transcription occurs in G1, then we may assume that *X(0)* ~ ½ *X_eq_* = ½ *b/d* in the beginning of G1 and

$$X_{t}=\frac{b}{d}-\frac{1}{2}\left(\frac{b}{d}\right)e^{-\text{dt}}$$ Eq. (2.2)

### TRR effect on mRNA abundance in one cell cycle

**Figure 1:**
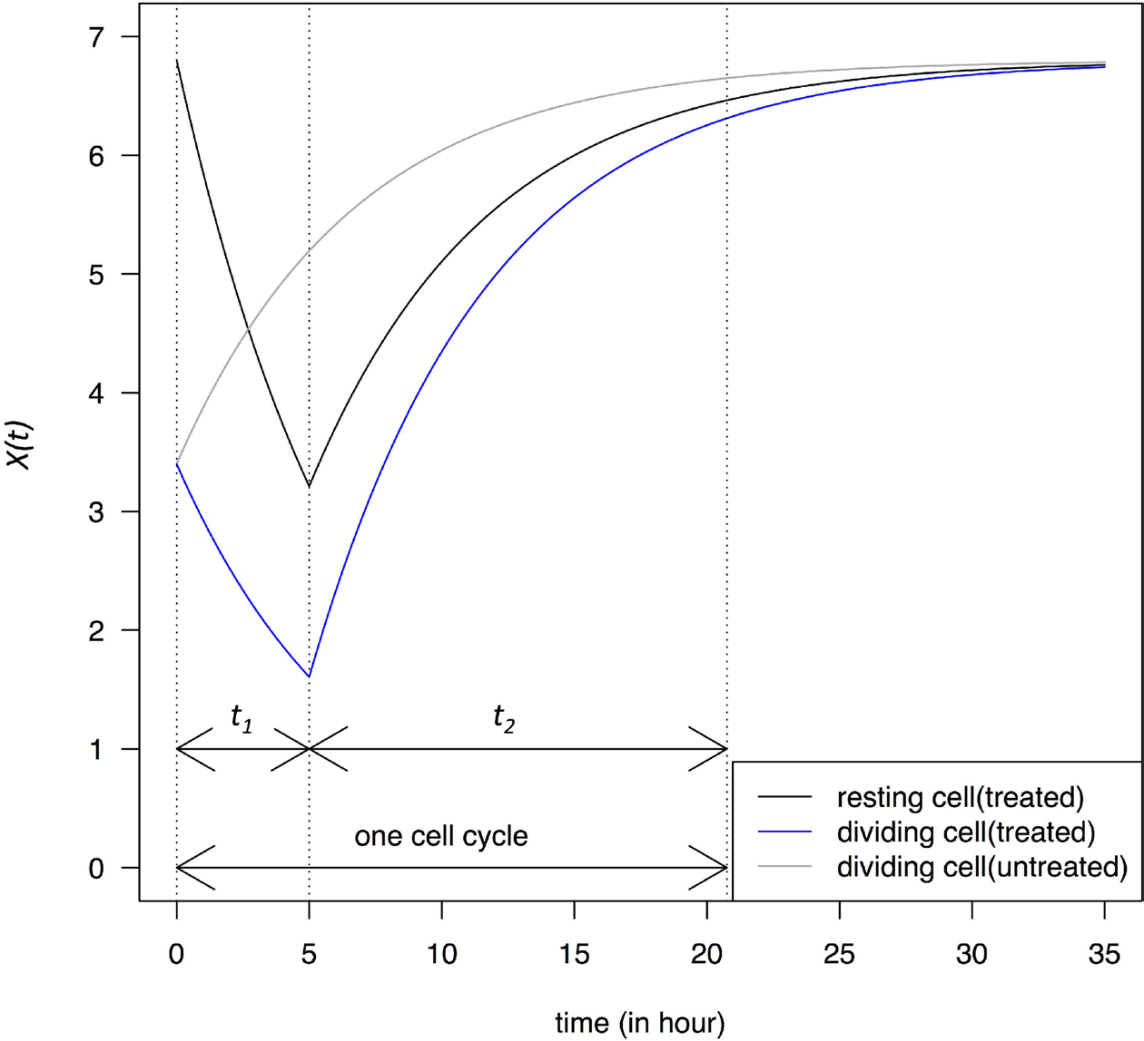
Change in transcript abundance per cell, *X(t)*, in a single cell cycle At time 0, dividing cells enter the G1 phase and *X(0)* = ½ *X_eq_*. Cells are either untreated or treated with a transcription-blocking agent for a period of *t_1_*, followed by a recovery phase that lasts for a period of *t_2_* until the end of the cell cycle. At the end of t_1_, *X(t)* of the resting cells is ½ *X_eq_*. The dynamics is described in Eqs. 2 – 2.2. Dividing cells are assumed to have a cell cycle time of 20 hours close to the limit of most cell lines.

When the transcription in resting cells is repressed by a drug such as actinomycin D, we reset *t* = 0 at application. *X(t)* then follows Eq. (2.1) until it decreases below a threshold that triggers cell death. In this case, cell death may be caused by p53-dependent apoptosis or other mechanisms shown to be operative [4–6, 15]. The repression (or blocking) phase lasts *t_1_* and the recovery phase lasts *t_2_*, as marked in Figure 1. It is necessary to block the transcription only briefly such that resting cells could rebound. We assume that *X(t) =* ½ *X_eq_* is a safe low bound for resting cells since dividing cells permit *X(t)* to reach this level. The trajectory within one cell cycle is shown in Figure 1. (Although resting cells do not actually go through cell cycles, the time is marked the same way as if cells are cycling.) *X(t)* fluctuates between *X_eq_* and ½ *X_eq_* and returns to near *X_eq_* in the time of one cell cycle.

*X(t)* changes in dividing cells under the same TRR schedule as in resting cells is also given in Figure 1 which shows the *X(t)* dynamics in cells that are at the M-G1 transition when the blocking takes place. Immediately after the M-G1 transition, untreated cells should be regaining transcripts that have been halved by cell division but, under drug treatment, *X(t)* would continue to decrease for the duration of *t_1_*. At the lowest point in this example, *X(t)* would be near ~ ¼ *X_eq_*, much lower than that of the resting cells at ½ *X_eq_*.

The recovery phase (*t_2_*) that follows is the main challenge of the TRR therapy. Starting from a lower transcript level at beginning of the treatment, dividing cells recover more rapidly than resting cells and continue to narrow the difference between the two types of cells. In the numerical calculation, the two types of cells will return to 95% and 93% of *X_eq_*, respectively in only one cell cycle. The faster recovery in cells with a lower *X(t)* is dictated by the term of *-dX(t)* in Eq. (1). We shall present strategies to address this challenge.

### TRR effect on mRNA abundance after multiple cell cycles

When the TRR treatment with the alternating blocking (*t_1_*) and recovery (*t_2_*) phases is applied through multiple cell cycles, the equilibrium dynamics can be modeled analytically. In each cell cycle, *X(t)* fluctuates between a high bound and a low bound, which quickly converge to the equilibrium, denoted *X_H_* and *X_L_*, respectively. For resting cells,

$$X_{H}=\frac{b}{d}\frac{1-e^{-dt_{2}}}{1-e^{-d(t_{1}+t_{2})}}$$ Eq.3

$$X_{\text{L}}=e^{-dt_{1}}X_{H}=\frac{b}{d}\frac{(1-e^{-dt_{2}})e^{-dt_{1}}}{1-e^{-d(t_{1}+t_{2})}}$$ Eq.4

For dividing cells, the corresponding values, *X_H_'* and *X_L_'*, are

XH'=bd1−e−dt21−12e−d(t1+t2) Eq.5

XL'=12e−dt1XH'=bd(1−e−dt2)e−dt12−e−d(t1+t2) Eq.6

**Figure 2:**
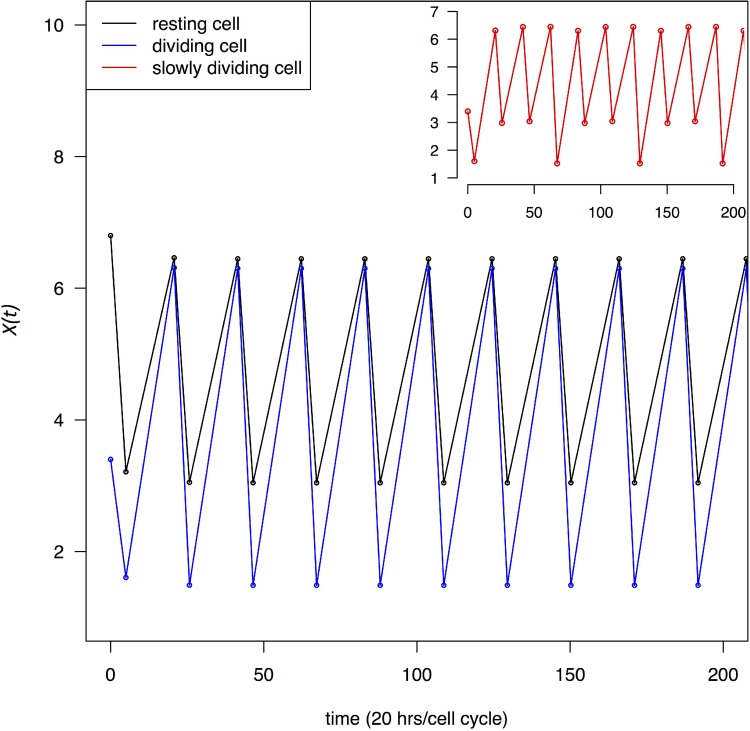
Change in transcript abundance per cell, *X(t)*, through multiple cell cycles Both dividing and resting cells are treated with a transcription blocking agent as in Figure 1. *X(t)* oscillates between a high and low value in each cycle which quickly converges to *X_H_* and *X_L_* defined by Eqs (3–6). Black and blue lines trace *X(t)* for resting and dividing cells respectively. In the inset, the red line trace cells that divide at 1/3 of the maximal rate.

The ratio of *X_L_'/ X_L_* is

$$RX_{L}=\frac{1-e^{-d(t_{1}+t_{2})}}{2-e^{-d(t_{1}+t_{2})}}$$ Eq. 7

which shows that the ratio is independent of *t_1_* and *t_2_* as long as *t_1_ + t_2_* is a constant. In this study, *t_1_ + t_2_* is the cell cycle time. In Figure 2, *X(t)* at the beginning and end of each recovery phase through multiple cell cycles is given. Given the rapidity with which *X(t)* approaches *X_H_* and *X_L_* (See Figure 2), Eqs 3–6 are sufficient to capture most of the dynamics. Figure 2 shows that TR depresses transcript abundance to a much lower level in dividing cells than in resting cells. We also assume that some cells may divide at a moderate rate, for example, at 1/3 of the maximal rate. The red line in the inset of Figure 2 marks the *X(t)* dynamics of cells that divide once every 60 hours.

We shall use *X_L_* as a gauge of how strongly cells are perturbed by the TRR treatment. Whereas rapidly dividing cells may die or stop dividing, the more moderately proliferative cells may be able to tolerate the TRR perturbation better since *X_L_* in such cells dips low less frequently. It is important to note that Figure 2 presents the “average dynamics” of all mRNAs. Among thousands of genes involved in this process, a small fraction of them may exert a fitness effect on the dividing cells and the large fluctuations in *X(t)* are unlikely to persist for long. Actual cancer cells may either stop dividing or, if they continue to divide, trigger apoptosis. The decision may depend on the mutations they carry. For example, in RB- and p53- cells, cell cycle progression may not be stopped at the check point and unhealthy cells may continue to divide leading to cell death [28].

### Efficacy of TRR as a function of blocking time (*t1*) in each cell cycle

**Figure 3:**
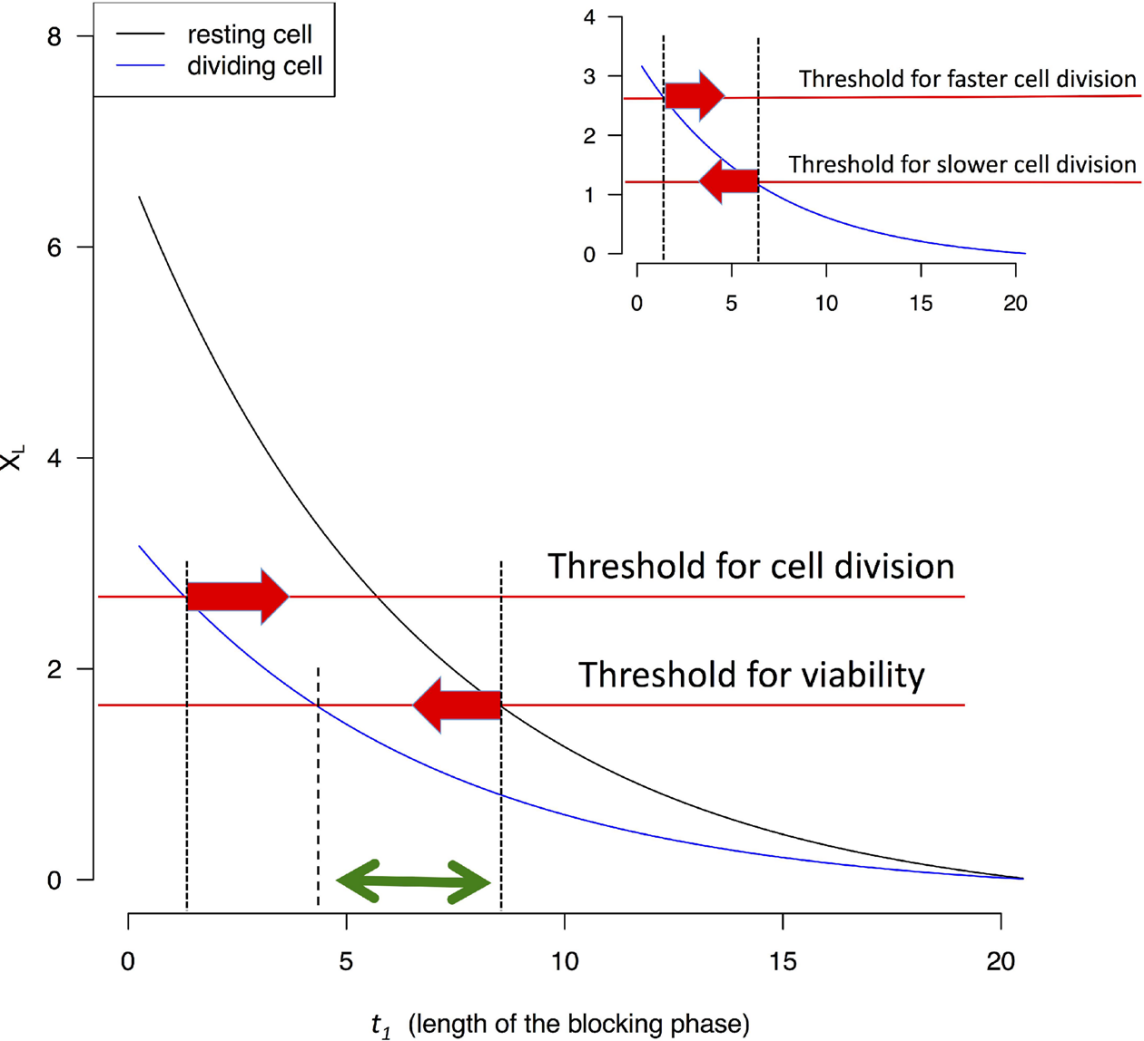
*X_L_* value (Eq. 4 and 6) as a function of the length of the blocking phase (*t*_1_) Two thresholds are assumed below which cells either do not divide or cannot survive. The two thresholds can be the same without affecting the qualitative conclusion. When *X_L_* drops below the threshold, cells are assumed to be unable to divide, or survive. The interval between the two red arrows indicates possible *t_1_* values that permit resting cells to survive but prevent non-resting cells to divide. The green arrow indicates a smaller interval for *t_1_* which would not permit dividing cells to survive (see text). In the inset, the comparison is between fast and slowly dividing cells. It is assumed that slowly dividing cells have a lower threshold for *X(t)* for cell survival since they are stressed much less often.

At the end of *t_1_*, cells are allowed to recover until the beginning of the next cell cycle. Figure 3 shows that *X_L_* decreases as *t_1_* increases for both proliferating and resting cells. By TRR, as by other cancer treatments, the treatment against proliferating cells also exerts a price on the resting ones. We assume that *X(t)* cannot sustain cell divisions if it deviates too far from *X_eq_*. In Figure 3, this is shown by the threshold (the upper red line). It also seems reasonable to suggest a lower threshold for the viability of resting cells (the lower red line). Between the two thresholds, there exists a time interval for *t_1_* (between the two red arrows, corresponding to 2–8 hrs in this example) which would stop cell proliferation but would leave resting cells to recover. It may also be possible to find a smaller interval for *t_1_*which would kill proliferating cells without killing resting cells (the green double-headed arrow of Figure 3).

Highly proliferative cells are stressed by low *X(t)*'s more frequently than moderately proliferative ones. Figure 2 inset gives an example of cells that are stressed only 1/3 as frequently as the fastest proliferating cells. It may therefore be possible that slowly-dividing may be able to tolerate lower *X_L_*'s (as they occur less frequently). In Figure 3 inset, the interval between the two red arrows indicates the window of *t_1_* in which blocking transcription can disrupt the more proliferative cells and spare the less proliferative ones. In contrast with TRR, drugs targeting DNA replication usually kill all cells that enter the cell cycle.

### Efficacy of TRR as a function of repression strength

For the TRR therapy to discriminate against the proliferating cells, relatively short blocking time with strong repression is recommended. We may ask if the complementary strategy of long blocking time with weak repression might also work. For this analysis, we introduce a new parameter α to designate the residual transcription under blocking, which ranges from the complete block of α = 0 to no blocking at α = 1.

For rest cells,

$$X_{H}=\frac{b}{d}\frac{1-e^{-dt_{2}}+\alpha (1-e^{-dt_{1}})e^{-dt_{2}}}{1-e^{-d(t_{1}+t_{2})}}$$ Eq.8

$$X_{\text{L}}=\frac{\alpha b}{d}-\left(\frac{\alpha b}{d}-X_{H}\right)e^{-dt_{1}}=\frac{b}{d}\frac{\alpha (1-e^{-dt_{1}})+(1-e^{-dt_{2}})e^{-dt_{1}}}{1-e^{-d(t_{1}+t_{2})}}$$ Eq.9

For cancer cells,

XH'=bd1−e−dt2+α(1−e−dt1)e−dt21−12e−d(t1+t2) Eq.10

XL'=αbd−(αbd−XH'2)e−dt1=bd2α(1−e−dt1)+(1−e−dt2)e−dt12−e−d(t1+t2) Eq.11

When α = 0, Eqs. 8 - 11 are reduced to Eqs. 3–6.

**Figure 4:**
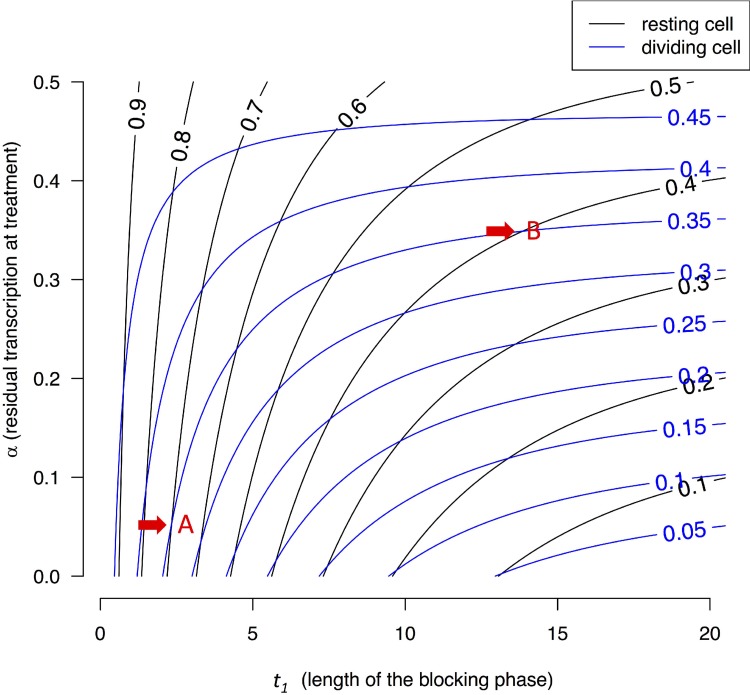
Contour lines of *X(t)/X_eq_* in resting and dividing cells *X(t)* is a function of both block time (*t_1_*) and blocking strength, expressed as the residual transcription rate (*α*) after blocking. Each point on the two-dimensional plane is associated with a pair of *X(t)* values. For example, point A, corresponding to a blocking time of *t_1_* = 2.5 hours and *α* = 0.05, is associated with (0.7, 0.35). In this case, resting cells are much less stressed by the treatment than dividing cells. In contrast, point B with *t_1_* = 12 hours and *α* ~ 0.35 is associated with *X(t)*'s of (0.4, 035), meaning both resting and dividing cells are comparably stressed. In general, lower left corners with short *t_1_* and low *α* values represent better treatment parameters.

Figure 4 presents the contour lines of *X_L_/X_eq_* for resting cells (black lines) and proliferating cells (blue lines) as the function of *t_1_* and α. The bottom portion of Figure 4 presents the dynamics under α ~ 0, equivalent to Figures 1, 2, 3. The lower left corner shows that black lines of high *X_L_/X_eq_* ratios (often > 0.5 for resting cells) and blue lines with low *X_L_/X_eq_* ratios (< 0.5) interleaf. In other words, intense blocking and short *t_1_* can lead to low *X(t)*'s in proliferating cells and high *X(t)*'s for resting ones. The upper right corner is of particular interest where blocking is weak but blocking time is long. This corner yields very different results from the lower left corner. Here, both blue and black lines of about 0.3–0.5 interleaf and there is no discrimination between proliferating and resting cells. In general, long blocking time (*t_1_*) blurs the differences between these two types of cells. This trend has been shown for strong blocking in Figures 1, 2, 3 and it is shown here for weak blocking.

## CONCLUSIONS AND DISCUSSION

The TRR strategy differs from the oncogene and transcription “addiction” hypotheses [29, 30]. The “addiction” hypotheses posit cancer-specific expressions that can be targeted for treatment. Nevertheless, the TCGA data show cancers to be highly heterogeneous at the molecular level and recent publications also report the low reproducibility of gene-based cancer findings [31–33]. We therefore assume a more general mechanism of global transcription repression.

TR agents such as actinomycin D are often potent against cancer cells [1–7, 9, 34]. For that reason, the success of TR therapy lies in the modulation of dose and duration that would spare normal cells but maintain the efficacy against cancer cells. To factor in toxicity reduction, we suggest TR be accompanied by a recovery phase in the same cell cycle as part of an expanded TRR strategy. Figure 4, incorporating intermediate repression, shows that the simple model can capture the general patterns of more complex interactions.

In realistic scenarios, the complex dynamics will depend on how the drug is systemically distributed and how it gets in the transcription complex. The mathematical theory suggests that the most effective strategy would be to administer the drug strongly but briefly, thus permitting a longer recovery time in the remainder of the same cell cycle. Given that the recovery phase demands rapid decline in drug concentration, local administration that permits diffusion to dissipate the drug may be a simple and effective strategy. The theory also provides the mathematical justification for the strategy of one single large dose over several smaller ones [2]. The theory is also relevant to the low-dose metronomic (LDM) chemotherapy [35], which administers drugs continuously but uses a reduced dose in each administration. In the absence of a quantitative framework, LDM studies often choose parameter values arbitrarily. The theory presented here will help to delineate the parameter space for the optimization of LDM.

TRR introduces a recovery phase (*t_2_*) that alternates with the blocking phase (*t_1_*) in the same cell cycle. The strategy may also be applicable to chemo-therapy that blocks DNA replication. In the DNA-based therapy, toxicity imposes a hefty cost on patients' health such that long term and repeated applications often cause severe problems. The theory of alternating the two phases in DNA-based therapies is beyond the scope of this study but it is conceivable that both phases would be somewhat longer than those for the transcription-based therapy.

Overall, transcription repression against mRNA transcripts can be effective but only when the application is carefully fine-tuned. Because the synthesis and degradation of mRNA transcripts are usually not affected by cell divisions, the separation between fast and slow cell divisions is narrow. In that regard, transcripts that are made at an accelerated rate in dividing cells would be much better targets. Hence, repression of rRNA synthesis may be a much more effective strategy. This topic will be addressed in a follow-up study.

Cancer therapy may be as much about the strategy of using existing drugs as developing new ones. TR and TRR therapies illustrate this point. With the delicate balancing of transcription repression and recovery, TRR may be able to discriminate among cells that have different needs for transcription. While highly proliferative cells generally have stronger needs, cancer and normal cells may also differ in their transcription dependence [29] irrespective of their rates of cell division. In conclusion, we suggest the TRR strategy as a first line of defense against the most highly proliferative cells. By stalling the rapid tumor growth, other therapies can be more carefully planned.
